# Acoustofluidic Diversity Achieved by Multiple Modes of Acoustic Waves Generated on Piezoelectric-Film-Coated Aluminum Sheets

**DOI:** 10.1021/acsami.4c06480

**Published:** 2024-08-15

**Authors:** Yong Wang, Xianbin Li, Hui Meng, Ran Tao, Jingui Qian, Chen Fu, Jingting Luo, Jin Xie, Yongqing Fu

**Affiliations:** † Department of Mechanical Engineering, Hangzhou City University, Hangzhou 310015, China; ‡ The State Key Laboratory of Fluid Power and Mechatronic Systems, Zhejiang University, Hangzhou 310027, China; § Faculty of Engineering and Environment, University of Northumbria, Newcastle upon Tyne NE1 8ST, United Kingdom; ∥ Anhui Province Key Laboratory of Measuring Theory and Precision Instrument, School of Instrument Science and Optoelectronics Engineering, Hefei University of Technology, Hefei 230009, China; ⊥ Shenzhen Key Laboratory of Advanced Thin Films and Applications, College of Physics and Optoelectronic Engineering, Shenzhen University Shenzhen 518060, China

**Keywords:** Acoustofluidic diversity, Multiple modes, Thin film acoustic waves, Fluidic actuation, Particle patterning

## Abstract

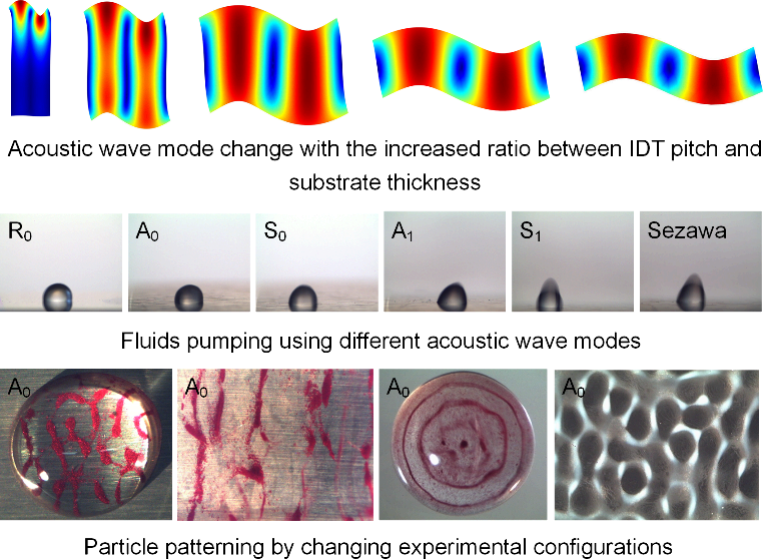

Excitation of multiple acoustic wave modes on a single chip is beneficial to implement diversified acoustofluidic functions. Conventional acoustic wave devices made of bulk LiNbO_3_ substrates generally generate few acoustic wave modes once the crystal-cut and electrode pattern are defined, limiting the realization of acoustofluidic diversity. In this paper, we demonstrated diversity of acoustofluidic behaviors using multiple modes of acoustic waves generated on piezoelectric-thin-film-coated aluminum sheets. Multiple acoustic wave modes were excited by varying the ratios between IDT pitch/wavelength and substrate thickness. Through systematic investigation of fluidic actuation behaviors and performances using these acoustic wave modes, we demonstrated fluidic actuation diversities using various acoustic wave modes and showed that the Rayleigh mode, pseudo-Rayleigh mode, and A_0_ mode of Lamb wave generally have better fluidic actuation performance than those of Sezawa mode and higher-order modes of Lamb wave, providing guidance for high-performance acoustofluidic actuation platform design. Additionally, we demonstrated diversified particle patterning functions, either on two sides of acoustic wave device or on a glass sheet by coupling acoustic waves into the glass using the gel. The pattern formation mechanisms were investigated through finite element simulations of acoustic pressure fields under different experimental configurations.

## 1 Introduction

Surface acoustic wave (SAW) technology has been demonstrated as an efficient and reliable method for manipulation of fluids and particles/cells, in either a digital format (e.g., using sessile droplets) or a continuous flow format (e.g., inside a polymer- or glass-based microchannel/chamber) with its attributes of biocompatibility, contactless and precise operation, and noninvasive features.^1−7^ Based on acoustic streaming and acoustic radiation effects induced by acoustic waves in the liquid,^8,9^ various microfluidic functions have been realized, including internal flowing/streaming,^10,11^ pumping,^12−14^ splitting/separation,^15,16^ jetting,^17−19^ nebulization,^20,21^ and particles/cells trapping,^22−24^ sorting,^25−27^ patterning,^28−30^ and alignment.^31−33^ Piezoelectric materials, either in their bulk forms (e.g., LiNbO_3_ or LiTaO_3_)^34−36^ or in thin film forms (e.g., ZnO or AlN)^37−39^ have been used for fabricating SAW-based acoustofluidic devices. Bulk ones, especially those based on LiNbO_3_ substrates, have been widely used due to their large piezoelectric constant, high electromechanical coupling coefficient (5%–11%) and high energy transduction efficiency.^13,37^ However, they are difficult to integrate with microelectronics for embedded control and signal processing, and also brittle and prone to fracture under a high radio frequency (RF) power and large thermoelectric shock.^38,39^ Moreover, it is difficult to excite multiple acoustic wave modes on LiNbO_3_ substrates once the crystal-cut and electrode’s pattern direction are defined. For example, a Rayleigh mode is generally generated on the 128° Y-cut LiNbO_3_ substrate, whereas a shear-horizontal SAW (SH-SAW) mode is usually excited on the 64° Y-cut LiNbO_3_ substrate.^7^ Multiple acoustic wave mode excitation on a single substrate or chip is beneficial for realizing diversified acoustofluidic functions.^40,41^ For instance, the Rayleigh wave is dominant for fluidic actuation due to its effective dissipation of acoustic wave energy into the liquid and the generation of an acoustic driving force, whereas the SH-SAW is suitable for biosensing in liquid environment on account of its slight signal attenuation in liquid. Additionally, excitation of multiple acoustic wave modes expands the range of available frequencies for various acoustofluidic manipulation, promotes the efficiency for microfluidic mixing, speeds up heat and mass transfer, and creates diversified flow fields by using multiple acoustic frequencies and vibration modes.^42,43^

Compared to their bulk counterparts, piezoelectric thin film acoustic wave technology has distinct advantages, in terms of device design’s flexibility (if considering in-plane isotropy of piezoelectric property), high breakdown voltage, low cost and easiness of fabrication and integration with other microelectronics.^44^ Besides, piezoelectric thin films, such as ZnO or AlN, can be easily deposited onto various substrates, including silicon,^45,46^ glass,^47,48^ polymers,^49,50^ and metals,^51−53^ thereby achieving various acoustic speeds, acoustic modes and applications such as the integration of fluidic actuation and biosensing functions on a single chip.^41,46^ In the past few years, various microfluidic actuation and particle/cell manipulation functions have been achieved using thin film acoustic wave devices fabricated on silicon, glass, and aluminum substrates with the Rayleigh mode. In our previous work, we have demonstrated microfluidic actuation/pumping functions using ZnO thin film acoustic waves on 50-μm-thick Al foil substrates with the zero-order mode (i.e., A_0_ and S_0_) of Lamb wave.^12,54^ Apart from the Rayleigh mode and zero-order mode of Lamb wave, there are also hybrid wave modes or higher-order modes of Lamb wave generation when the ratio between interdigital transducer (IDT) pitch/wavelength and substrate thickness is within an appropriate range.^42,55^ In addition, the substrate thickness plays an important role in fluidic actuation performance, as a much flexible device tends to generate a macroscopical substrate deformation during the actuation, resulting in poor fluid actuation performance. Until now, acoustofluidic behaviors (including fluidic pumping and particle patterning) under multiple acoustic wave modes, IDT pitches, and substrate thicknesses have not been systemically investigated, but these are important for realizing a diversity of acoustofluidic functions. For example, the selectively excited acoustic wave modes gain a good fluid actuation performance, the balance between the device flexibility and actuation performance maintains a good fluidic actuation performance for flexible devices, and the expansion of acoustic frequencies and resonances generate various flowing behaviors and particle patterns for multiple biological applications.

In this paper, we fabricated ZnO thin film acoustic wave devices with wavelengths varied from 100 μm to 1100 μm on Al sheet substrates with two thicknesses (i.e., 200 μm and 600 μm). We have achieved various acoustic wave mode excitations on ZnO-thin-film-coated Al sheets, including the Rayleigh, Sezawa, hybrid, and Lamb waves by varying the ratios between IDT pitch and substrate thickness. We systematically investigated fluidic actuation behaviors and performance using the generated acoustic wave modes and analyzed the actuation mechanism of the microfluidic behaviors. Moreover, we demonstrated diversified particle patterning abilities either on two sides (i.e., top and backside) of the Lamb wave device or on the glass sheet (by coupling of acoustic wave into the glass using the ultrasonic gel) with different orders of Lamb waves. We analyzed the formation mechanisms of particle patterns through finite-element analysis (FEA). Our work shows great potential for establishing diversified acoustofluidic platforms using multiple modes of acoustic waves and different experimental configurations.

## 2 Materials and Methods

ZnO thin films with a thickness of ∼5 μm were deposited onto commercially available Al sheet substrates (Metal Sheets Limited, U.K.) with thickness of 200 and 600 μm using a direct current (DC) magnetron sputtering system (Model NS3750, Nordiko). A zinc target with a purity of 99.99% was used for film deposition. The deposition was performed with a DC power of 400 W, a chamber pressure of ∼3 mTorr, and an Ar/O_2_ gas flow ratio of 3/10 sccm. The distance between the zinc target and the sample holder was 20 mm, and the sample holder was rotated to obtain uniform ZnO thin films. Crystal orientation of the deposited ZnO thin film on the Al sheet substrate was analyzed by using X-ray diffraction (XRD) (Model D5000, Siemens) with Cu Kα radiation (λ = 1.5406 Å). Cross-sectional morphology of the ZnO thin film was observed using a scanning electron microscopy (SEM) system (Model S-4100, Hitachi). The characterization results of ZnO thin film on Al sheet substrate are shown in Figure S1 in the Supporting Information. IDT electrodes of acoustic wave devices were patterned by evaporating a layer of 150-nm-thick Al on a ZnO/Al sheet substrate. There were 60 pairs of finger electrodes on each IDT with an acoustic aperture of 5 mm and wavelengths that varied from 100 to 400 μm, as shown in Figures S2(a) and S2(b). For the acoustic wave device with the wavelength of 1100 μm, the number of finger pairs was 20 and the acoustic aperture was 15 mm, as shown in Figure S2(c). The reflection spectra (*S*_11_) of the acoustic wave devices were measured using an RF network analyzer (Agilent, Model E5061B).

To understand acoustic wave vibration modes and acoustic pressure fields under different experimental configurations, FEA simulations were performed using COMSOL Multiphysics (6.1) software with solid mechanics, electrostatics, and pressure acoustic modules. For simulations of acoustic wave modes, a simplified two-dimensional (2D) model with one pair of IDT electrodes and infinite boundary conditions was used. The schematic of 2D model and meshing of 2D model are shown in Figures S3(a) and S3(b), respectively. Here, the thickness of ZnO thin film was 5 μm, and the electrode thickness was 150 nm. The IDT pitch was varied from 100 to 1100 μm, and the Al sheet substrate thickness was set as 200 and 600 μm, respectively. A polarization voltage of 1 V was assigned to one of the IDT electrodes, while another was assigned to be ground. To really reflect the wave vibration mode, the bottom boundary was set to be free, whereas the boundaries of lateral walls were set as periodic, as shown in Figure S3(b). Triangular meshes was used to mesh the 2D model with the maximum element units ranging from 10 to 30 μm and the minimum element units varied from 0.1 to 0.3 μm, depending on the IDT pitch and substrate thickness.

For simulations of acoustic pressure fields, a 3D model of acoustic wave device composed of the Al electrode, Al substrate, and ZnO thin film layer was built, as shown in Figure S3(c). Here, the electrode thickness was 150 nm, the thickness of the Al substrate was 200 μm, the thickness of ZnO thin film was 5 μm, and the IDT pitch/wavelength was 1100 μm with an acoustic aperture of 8 mm. The diameter of droplet model was 3.2 mm with a height of 1.2 mm. A PDMS model with dimensions of 3.5 mm (*W*) × 1.5 mm (*H*) × 4 mm (*L*) and wall thickness of 0.4 mm was used. The thick of glass model was 200 μm. Free tetrahedral meshes were used for meshing the 3D model with the maximum and minimum mesh size limited to 300 and 162 μm, respectively, as illustrated in Figure S3(d). Mesh size is a compromise between computational accuracy and computational cost. Therefore, the maximum element growth rate was set as 1.6, thereby avoiding too small mesh and inverted surface mesh. For simulations of acoustic pressure fields on the device surface, the sessile droplet or PDMS chamber containing the liquid was directly placed on the device surface, then the acoustic wave was coupled into the liquid. Whereas, for the acoustic pressure field simulation on the glass sheet, the acoustic wave was first coupled into the glass, and then the propagating acoustic wave on the glass was further coupled into the liquid. For each pair of IDT electrodes, a polarization voltage of 10 V was assigned to one of the electrodes, while the other one was assigned to be ground. Finally, acoustic pressure fields under different experimental configurations were simulated in the frequency domain analysis using the corresponding frequency values obtained from the eigenfrequency analysis. The detailed material parameters were obtained from the literature.^41,55^

For performing acoustofluidic tests, the acoustic wave device was put on an aluminum alloy test holder to minimize potential acoustic heating effect.^38^ An RF signal was generated using a signal source (RIGOL, DSG 815) and amplified using a power amplifier (Aigtek, Model ATA-1222A) before being input into the IDT electrodes (Figure S4). The input RF power was measured using a feedthrough-type power meter (Model SHX-200W-3601-HUV, SHX). The used RF power range for particle patterning was varied from 0.2 to 1 W, and the RF power range for fluidic actuation was varied from 1 to 30 W. For fluidic actuation tests, a layer of 200-nm-thick CYTOP (Asahi Glass Co., Tokyo, Japan) was coated on the surface of the acoustic wave device to make the surface hydrophobic. After the hydrophobic treatment, the droplet’s contact angle on the ZnO thin film surface is ∼109°. To visualize particle patterns induced by the acoustic waves, both silica (with a diameter of 5 μm) and polystyrene (with a diameter of 10 μm) were used, and they were added into the deionized (DI) water to prepare different particle solutions, respectively. Then, the particle solution was deposited dropwise on the device’s top surface or injected into the rectangular polydimethylsiloxane (PDMS) chamber bonded onto the top or backside of the device to observe the formation of particle patterns. The PDMS chamber with dimensions of 3.5 mm × 1.5 mm × 4 mm (*W* × *H* × *L*) was fabricated by using the standard soft photolithography process. A piece of glass with a thickness of 50 μm was put on top of the PDMS chamber to avoid the evaporation and also induced the wave reflection. The particle solution was also dropped on a glass sheet (200 μm thick) or injected into the gap between two pieces of glass to observe the formed particle patterns under acoustic wave agitations. Here, an ultrasonic gel (AQUASONIC100) was used to effectively transfer the acoustic wave energy into the glass from the acoustic wave device. The acoustic wave mode, which was linked with a specific wavelength, could be selectively excited by applying the corresponding frequency at the resonant peak. A CCD camera (NPX-GS130UM, 100 frames/s) was used to capture the motions of the fluid and particle trajectories.

## 3 Results and Discussion

Previous studies have shown that the ratio *r* (*r* = λ/*h*) between the IDT pitch λ and substrate thickness *h* played an important role in exciting various acoustic wave modes.^42,55^ When the ratio *r* was ≪1, the Rayleigh mode was dominant, whereas the Lamb wave mode was dominant when the ratio *r* was ≫1. Both the Rayleigh mode and the Lamb wave mode would be hybridized together when the ratio *r* was ∼1. Figure S5 shows FEA simulation results of acoustic wave modes and experimentally measured reflection spectra for ZnO thin film acoustic wave devices fabricated on a 600-μm-thick Al sheet with wavelengths varied from 100 to 400 μm. Here, the acoustic wave mode at the resonant peak of reflection spectrum was determined by comparing the resonant peak frequency with the simulated frequencies at different acoustic wave modes. We have found that when the device wavelengths were 100 and 200 μm, in which the ratio *r* was much smaller than one, both the Rayleigh mode and Sezawa mode were excited. As the device wavelengths were increased to 300 and 400 μm, hybrid pseudo-Rayleigh mode and pseudo-A_0_ mode of Lamb wave were obtained based on the results from both FEA simulation and experiment measurement. Whereas from the experimental results, we have not found distinct resonant peak near the frequency of simulated pseudo-S_0_ mode. Here, the pseudo-Rayleigh mode, pseudo-A_0_ mode, and pseudo-S_0_ mode are defined as hybrid acoustic modes, which have shown the similar vibration patterns to those of the fundamental Rayleigh mode, A_0_ mode and S_0_ mode, respectively.

To further expand the generated acoustic wave modes, the acoustic wave devices were fabricated on a thinner substrate (200 μm). Figure S6 shows FEA simulation and experimental measurement of wave vibration modes for ZnO thin film acoustic wave devices fabricated on a 200-μm-thick Al sheet with wavelengths varied from 100 to 1100 μm. When the device wavelength was 200 μm and the ratio *r* was 1, a hybridization between the Rayleigh mode and A_0_ mode of the Lamb wave was observed. Additionally, both the pseudo-S_0_ mode and the Sezawa mode were observed from FEA simulation and experiment measurement. As the ratio was further increased to 1.5 and 2 (corresponding to device wavelengths of 300 and 400 μm), the first-order mode (i.e., pseudo-A_1_ and pseudo-S_1_) of Lamb wave was observed, apart from the generated zero-order mode (i.e., A_0_ and S_0_). Moreover, there was a hybrid S_1_ mode and Sezawa mode for the acoustic wave device with a wavelength of 300 μm. When the ratio *r* was increased to 5.5 with a wavelength of 1100 μm, the acoustic wave mode was a pure Lamb wave, i.e., zero-order and higher-harmonic modes. Therefore, as the ratio *r* was increased from 0.17 to 5.5, the acoustic wave mode was changed from the Rayleigh mode (Sezawa mode, also called the two-order Raleigh wave) to hybrid mode and then to Lamb wave mode.

### 3.1 Fluid Actuation Using Multiple Modes of Acoustic Waves

**Figure 1:**
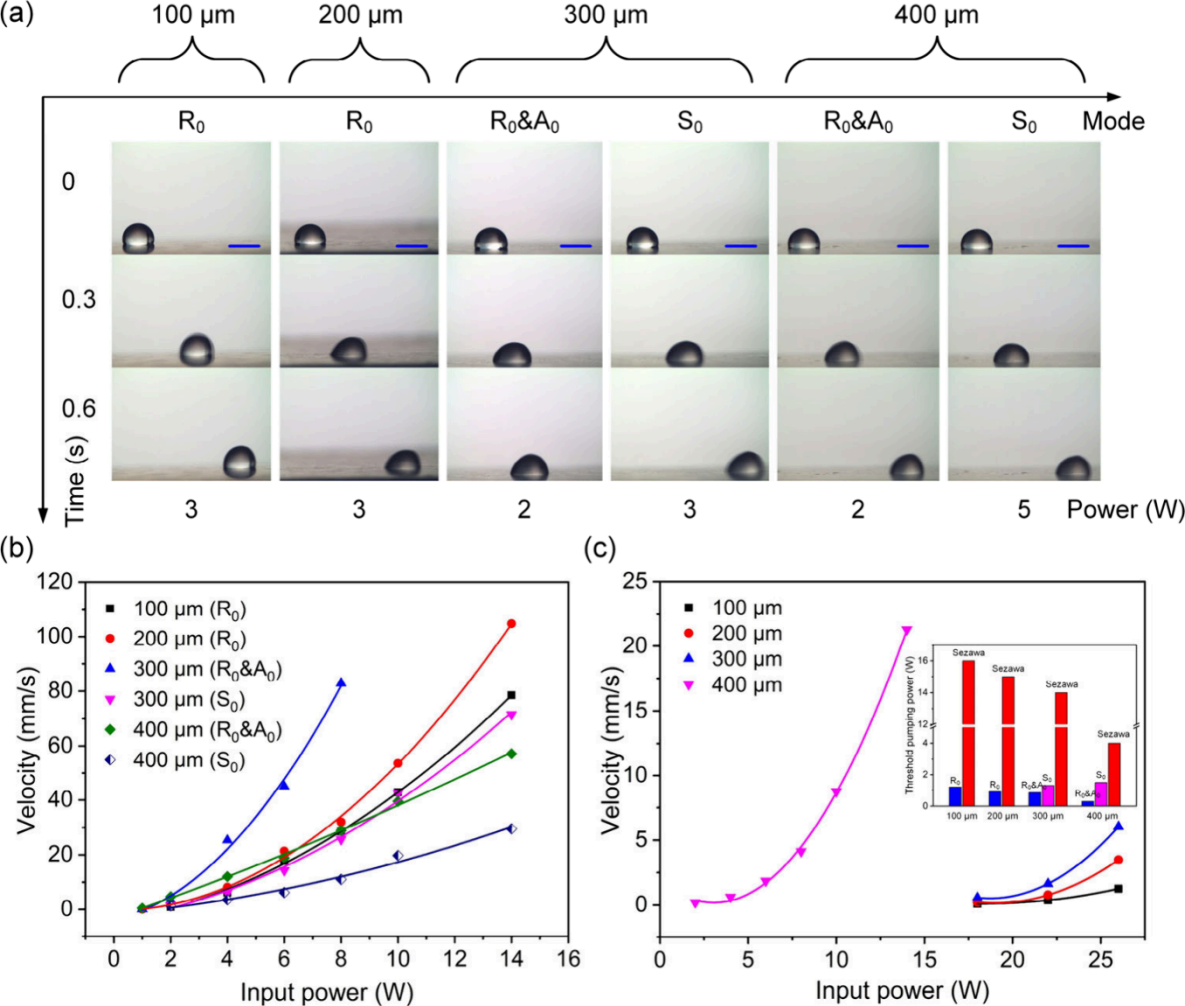
(a) Droplet pumping images using ZnO thin film acoustic wave devices (with wavelengths varied from 100 to 400 μm) fabricated on a 600-μm-thick Al sheet substrate with different acoustic wave modes. All scale bars are 1 mm. (b) Droplet average pumping velocities for ZnO thin film acoustic wave devices fabricated on 600-μm-thick Al sheet substrates with different acoustic wave modes under different input powers. (c) Droplet average pumping velocities for ZnO thin film acoustic wave devices fabricated on 600-μm-thick Al sheet substrates using the Sezawa mode under different input powers, the inset shows threshold pumping power (the minimum power to initiate the droplet) for different wavelength acoustic wave devices using different acoustic wave modes.

The microfluidic behaviors induced by the acoustic waves are determined by the input RF power. When the input RF power was relatively low (e.g., tens of milliwatts), acoustic streaming effects within the droplet were observed.^37^ As the input RF power was increased to the level of a few watts, the droplet vibration, pumping and even jetting could be achieved.^56^ Here, we focus on droplet pumping or transportation behaviors by using different acoustic wave modes. Figure 1a shows droplet pumping phenomena on a 600-μm-thick Al sheet substrate using various acoustic wave modes under different wavelengths or IDT pitches. For a single-pitched IDT, the specific acoustic wave mode was selectively excited by using the corresponding frequency. For the Rayleigh mode and pseudozero-order mode (i.e., pseudo-A_0_ and pseudo-S_0_) of Lamb wave, the droplet motion on the Al sheet is a combination of rolling and sliding (Movie S1). This is mainly due to a large diffraction angle (∼32°) between the acoustic wave and the liquid, which is often called Rayleigh angle (θ_*R*_ = sin^–1^(*C*_F_/*C*_S_), where *C*_F_ is the sound speed in a liquid, *C*_S_ is the acoustic wave propagation speed in the substrate) for the Rayleigh wave. Whereas, for the Sezawa wave mode, the droplet movement tends to be a combination of jumping and sliding (Movie S2), due to the high acoustic speed of the Sezawa wave, thus quickly dissipating acoustic energy. Also, the smaller value of diffraction angle (∼16°) could easily lift up the droplet.

**Figure 2:**
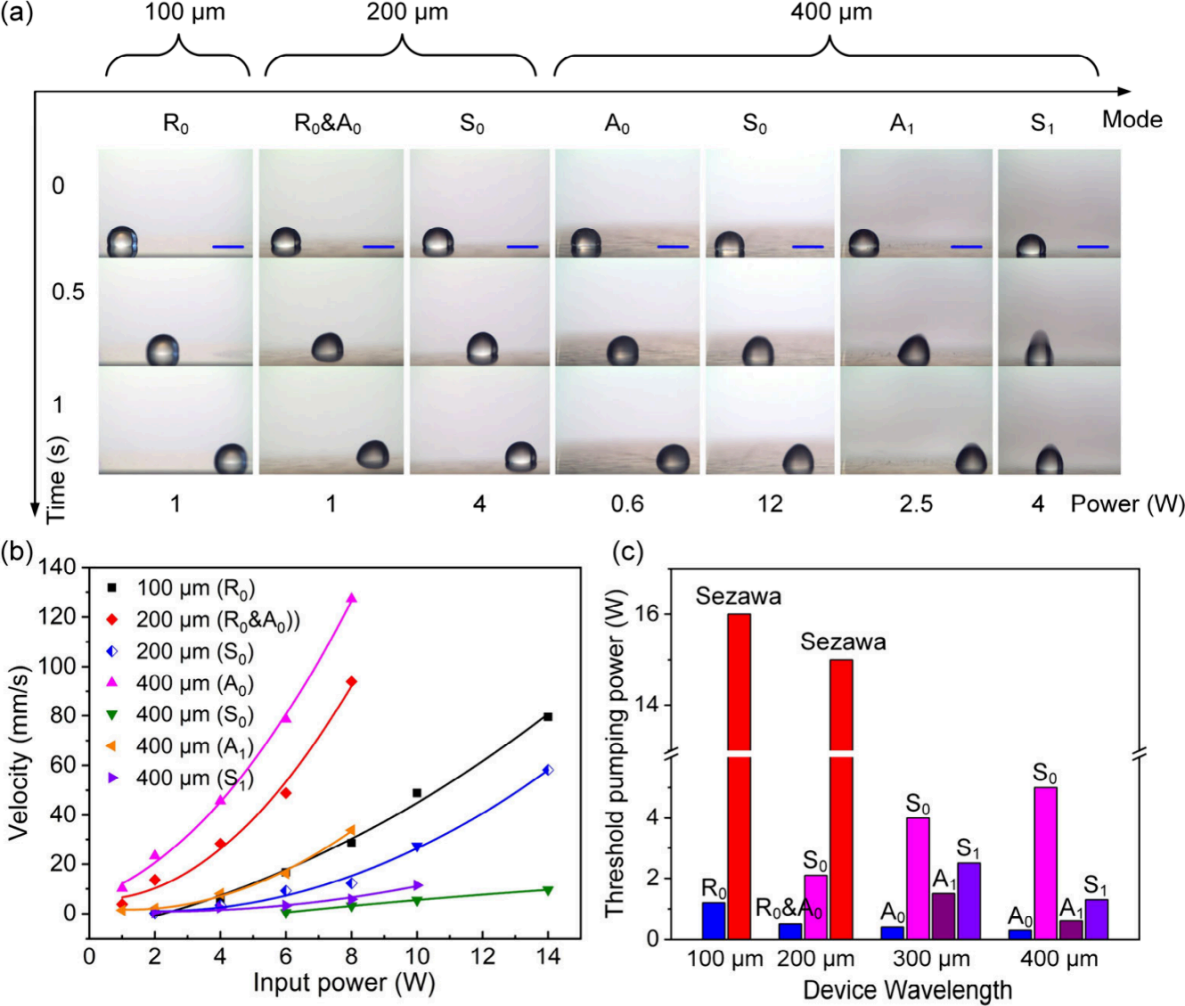
(a) Droplet pumping images using ZnO thin film acoustic wave devices (with wavelengths of 100, 200, and 400 μm) fabricated on a 200-μm-thick Al sheet substrate with different acoustic wave modes. All scale bars are 1 mm. (b) Droplet average pumping velocities for ZnO thin film acoustic wave devices fabricated on a 200-μm-thick Al sheet substrate with different acoustic wave modes under different input powers. (c) Threshold pumping powers for different wavelength acoustic wave devices using different acoustic wave modes.

The droplet’s average pumping velocities under different acoustic wave modes were measured, with results shown in Figures 1b and 1c. As the input RF power was increased, the droplet pumping velocity was increased. For acoustic wave devices with wavelengths of 100–300 μm, the droplet pumping velocity using the Rayleigh mode or pseudo-A_0_ mode of Lamb wave was increased with the device wavelength, because a large wavelength can result in a long acoustic wave penetration length (i.e., the interaction distance between the acoustic wave and the liquid),^57^ thereby improving fluidic actuation efficiency for this given droplet size. For the acoustic wave device with a wavelength of 400 μm, the droplet pumping velocity was slightly smaller than that of the 300 μm one using either pseudo-A_0_ mode or pseudo-S_0_ mode. This is mainly because when the ratio *r* is closer to 1, the acoustic wave mode tends to change from the Rayleigh mode to the Lamb wave mode. The Rayleigh wave are propagating more near the device’s surface, thereby having a high energy conversion efficiency. In addition, fluidic pumping performance using the Rayleigh mode or pseudo-A_0_ mode is distinctly better than that of the pseudo-S_0_ mode and Sezawa mode. For the Sezawa mode, the droplet pumping performance is improved with an increase of device wavelength, as shown in Figure 1c. This is because as the ratio *r* is increased, the Sezawa mode tends to convert into the A_1_ mode of the Lamb wave, and the pumping performance using the A_1_ mode is much better than that of the Sezawa mode, as demonstrated in Figure 2c.

To further investigate effects of acoustic wave modes/orders on fluidic actuation behaviors and performance, we selected the acoustic wave devices with an Al substrate thickness of 200 μm. Figure 2a shows droplet pumping behaviors using ZnO thin film acoustic waves on a 200-μm-thick Al sheet substrate with different acoustic wave modes. For different cases of Rayleigh mode, hybrid mode (e.g., pseudo-A_0_ and pseudo-S_0_), and zero-order mode (i.e., A_0_ and S_0_) of Lamb wave, the droplet movement on the Al sheet substrate was found to be a combination of rolling and sliding (Movie S3). The droplet pumping behaviors shown in Movies S1 and S3 are similar, indicating that fluidic actuation behaviors are mainly determined by the acoustic wave modes rather than the substrate thickness. Because as long as the used acoustic wave mode is the same, the droplet motion on the Al sheet looks similar, no matter how thick the Al sheet is. Whereas for first-order mode (i.e., A_1_ and S_1_) of Lamb wave case, the droplet movement was dominated by jumping and sliding (Movie S4). The droplet pumping behaviors using the first-order mode of Lamb wave were similar to those of the Sezawa mode, which is attributed to a high acoustic speed of the first-order mode of Lamb wave and a small diffraction angle (∼16°), which tends to lift up the droplet. Another possible reason is that the surface vibration mode of S_1_ mode is partly similar to that of the Sezawa mode (Figure S6).

Figure 2b shows droplet average pumping velocities for different wavelength acoustic wave devices with different modes. The droplet pumping velocities using the Rayleigh mode, A_0_ mode, or A_1_ mode were much higher than those of the S_0_ mode and S_1_ mode at the same input RF power. In addition, the threshold pumping powers using the Rayleigh mode, A_0_ mode, or A_1_ mode were much smaller than those of Sezawa mode, S_0_ mode, and S_1_ mode, as shown in Figure 2c, indicating that the Rayleigh mode and antisymmetric mode (e.g., A_0_ and A_1_) of Lamb wave present better pumping performance. Moreover, the pumping performance using the zero-order mode (i.e., A_0_ and S_0_) of the Lamb wave was generally better than that of the first-order mode (i.e., A_1_ and S_1_), revealing that the lower-order mode acoustic waves generally show a better fluid actuation performance.

**Figure 3:**
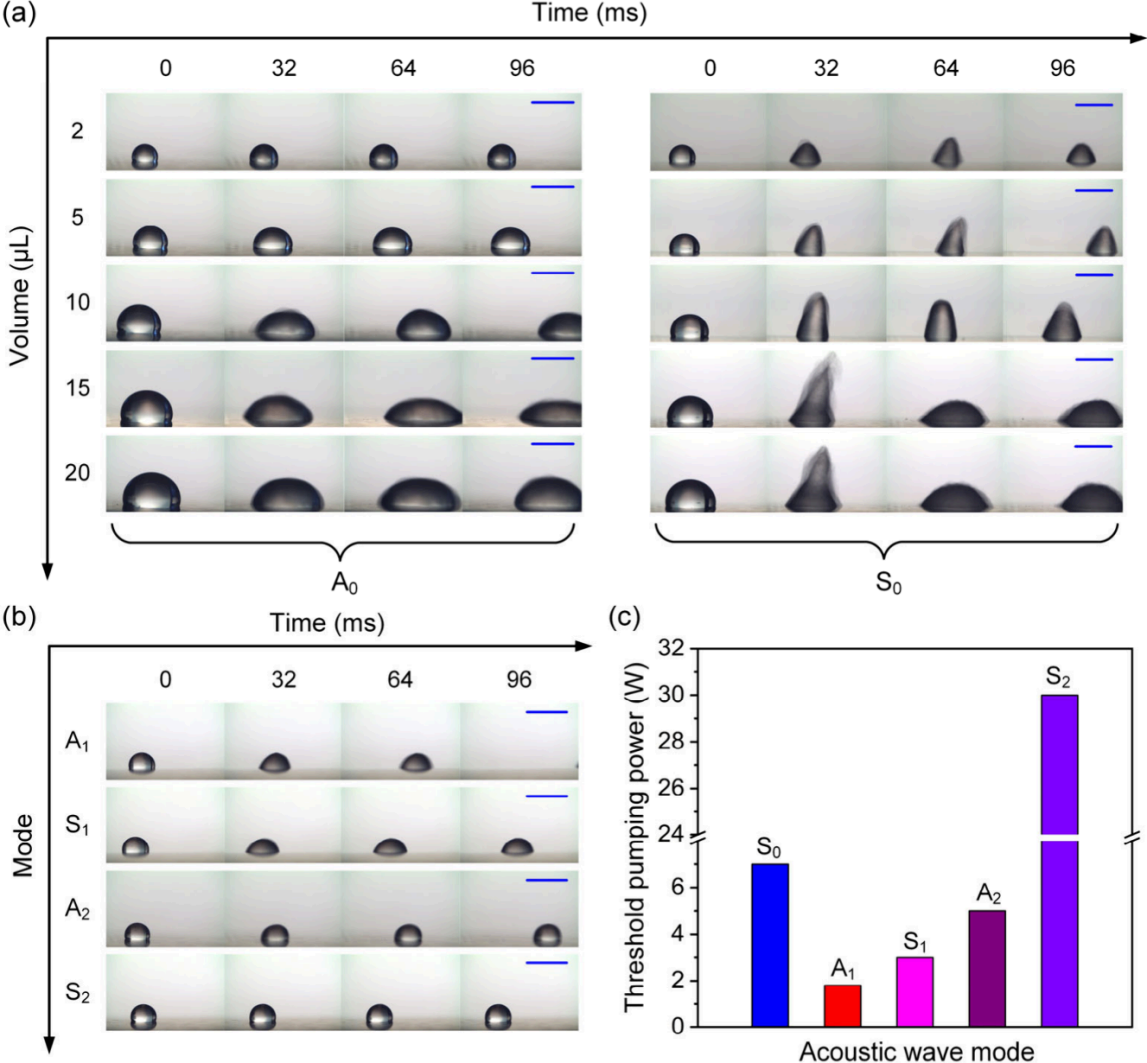
(a) Droplet pumping images with different volumes (2 to 20 μL) using ZnO thin film acoustic waves on a 200-μm-thick Al sheet substrate with the A_0_ mode and S_0_ mode. The wavelength of the acoustic wave device is 1100 μm. (b) Droplet pumping images using ZnO thin film acoustic waves on a 200-μm-thick Al sheet substrate with the A_1_ mode, S_1_ mode, A_2_ mode, and S_2_ mode. (c) Threshold pumping powers for ZnO thin film acoustic waves on a 200-μm-thick Al sheet substrate using different acoustic wave modes. All scale bars are 2 mm.

When the ratio *r* is increased to 5.5, i.e., with a wavelength of 1100 μm, a zero-order and higher-order mode (e.g., A_1_ and S_1_, A_2_ and S_2_) of Lamb waves would be excited. Here, we further investigated fluidic pumping behaviors and performance using different orders and modes of the Lamb wave. Figure 3a shows droplet pumping behaviors using a zero-order mode (i.e., A_0_ and S_0_) of the Lamb wave. For the A_0_ mode, there was a threshold value for the droplet volume or size (∼5 μL) to drive the droplet, above which, the droplet could be actuated forward (Movie S5). Otherwise, the droplet could only be horizontally stretched (side view; Movie S6). This is because when the device wavelength is larger than or near the droplet size, part of acoustic wave will penetrate the droplet and generate a strong wave reflection at the back of the droplet,^33^ thereby preventing the droplet to move forward. For the S_0_ mode, there was no obvious size limitation to actuate the droplet, and the droplet tended to jump or jet under the actuation of an acoustic wave. We also studied the droplet pumping using higher-order modes (e.g., A_2_ and S_2_), with the results shown in Figure 3b. The obtained threshold pumping powers using the higher-order mode were generally much higher, as shown in Figure 3c. For example, for the S_2_ mode of the Lamb wave, even though the input RF power was increased to tens of watts, the droplet was still difficult to be transported forward. In brief, the fluidic pumping performance using the antisymmetric modes (e.g., A_1_ and A_2_) of Lamb wave is generally better than that of symmetric modes (e.g., S_1_ and S_2_).

Although different acoustic wave modes may bring different fluid actuation behaviors, for practical application of acoustofluidic actuation devices, we should focus more on the fluidic actuation performance. Therefore, effects of IDT pitch/wavelength, substrate thickness, acoustic wave mode, and droplet size on fluidic actuation performance need to be carefully considered. For example, the increase of the device’s wavelength or IDT pitch can improve fluidic actuation performance to some extent, because the increased interaction distance between the acoustic wave and the liquid enhances the energy conversion efficiency. However, when the device wavelength is increased to nearly or slightly above the substrate thickness (i.e., the ratio *r* is near or larger than 1), there is an acoustic wave mode transition between the Rayleigh wave and Lamb wave, thereby resulting in different fluidic actuation performances. Additionally, the substrate thickness also has an important influence on fluidic actuation performance. A more flexible/bendable acoustic wave device may produce an obvious substrate deformation or significant damping effect during the actuation,^54^ thereby causing acoustic energy dissipating into the substrate and significant acoustic heating effect. Furthermore, for a given size droplet, when the device wavelength was increased to nearly or above the droplet size, the droplet pumping performance using the low-frequency zero-order mode of the Lamb wave may not be good due to significant wave reflection at the droplet boundary.

### 3.2 Particles Patterning Using Multiple Modes of Acoustic Waves

Apart from fluidic actuation functions, acoustic waves can also be used for manipulating particles within the fluid. The gravity effect on microparticles in liquid is commonly ignored due to their small sizes. Therefore, the particles suspended in the fluid mainly experience two acoustic forces: the acoustic radiation force and the drag force resulting from acoustic streaming.^43,58,59^ The acoustic radiation force applied to the particle can be expressed as^45^
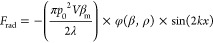
1
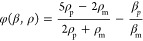
2
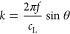
3where *F*_rad_ corresponds to the acoustic radiation force, *p*_0_ is the acoustic pressure, *V* is the particle volume, β_m_ is the compressibility of medium, β_p_ is the compressibility of particles, λ is the wavelength, φ(β,ρ) is the acoustic contrast factor, *k* is the wavenumber, *x* is the distance of the particle from the pressure node, ρ_p_ is the particle density, ρ_m_ is density of the medium, *f* is the driving frequency, *c*_L_ is the longitudinal wave velocity, and θ is the diffraction angle.

The acoustic streaming induced drag force acting on a particle can be calculated as follows:^60^
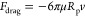
4where μ is the fluid dynamic viscosity, *R*_p_ the particle radius, and *v* the relative velocity between the fluid and particles.

In general, the acoustic radiation force is the main driving force for particle manipulation, whereas the acoustic streaming induced drag force is mostly regarded as the disturbance. Previous studies showed that there was a threshold particle size to balance these two forces, above which, the acoustic radiation force became dominant.^61^ At a driving frequency of 2 MHz, the threshold particle size is ∼2.6 μm, and this size is proportional to *f*_–1/2_. For a particle patterning test, here, a ZnO-thin-film-based Lamb wave device fabricated on a 200-μm-thick Al sheet with wavelength of 1100 μm was used. The frequency range of different orders of the Lamb wave is from 1.39 to 11. Two MHz, corresponding to a threshold particle size ranging from 3.1 to 1.1 μm. Therefore, when the particle size is larger than 3.1 μm, the particle motion within the fluid will be mainly determined by the acoustic radiation force. Here, the used particle size is 5 and 10 μm, respectively. Thus, when the applied RF power is relatively low (neglecting acoustic streaming effects), under the actuation of acoustic radiation force, the particles will be accumulated at the pressure nodes corresponding to acoustic pressure fields,^62^ thereby generating various particle patterns.

**Figure 4:**
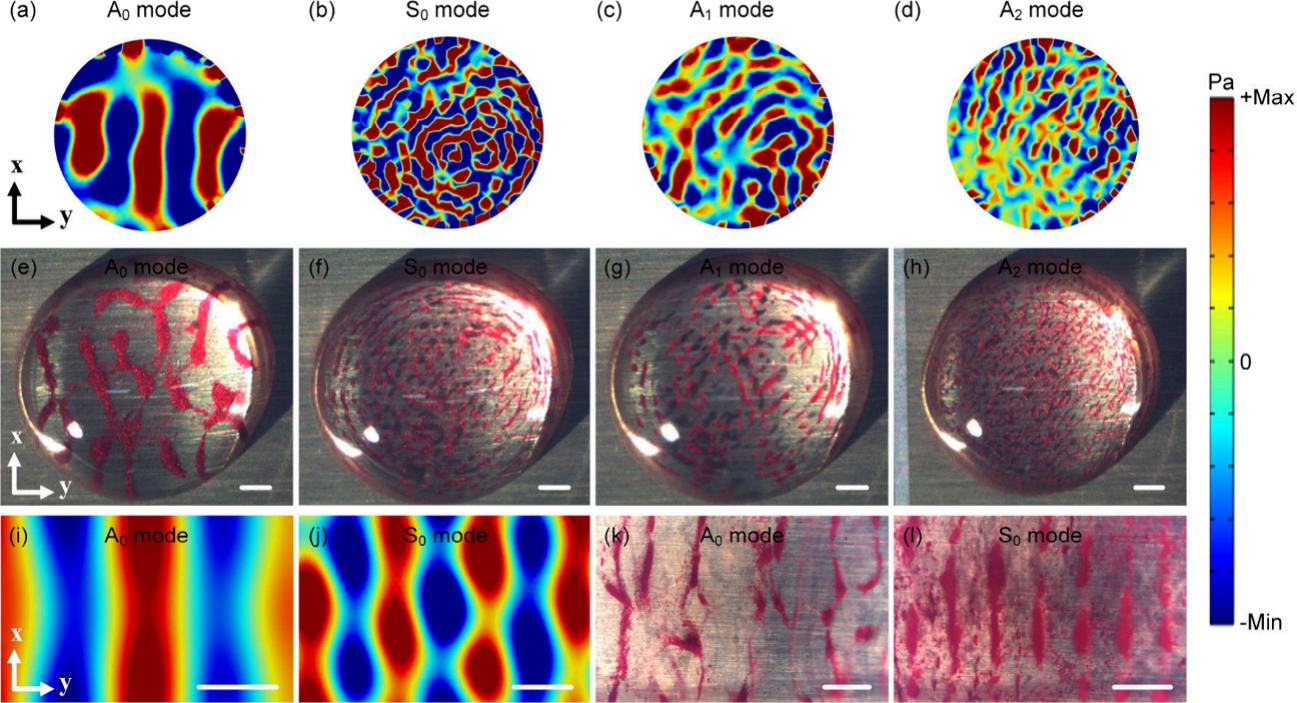
Simulation of acoustic pressure fields within the droplet positioned on the device surface using different modes/orders of Lamb wave: (a) A_0_ mode (1.39 MHz), (b) S_0_ mode (4.81 MHz), (c) A_1_ mode (4.01 MHz), and (d) A_2_ mode (6.83 MHz). Polystyrene particle (diameter of 10 μm, red color) patterning within the droplet (50 μL) positioned on the device surface using different modes/orders of Lamb wave, (e) A_0_ mode (1.38 MHz, 0.2 W), (f) S_0_ mode (4.86 MHz, 0.3 W), (g) A_1_ mode (4.01 MHz, 0.3 W), and (h) A_2_ mode (6.81 MHz, 0.5 W). Simulation of acoustic pressure fields inside the PDMS chamber positioned on device surface (in front of IDT) using (i) the A_0_ mode (1.39 MHz) and (j) the S_0_ mode (4.81 MHz). Polystyrene particles (diameter of 10 μm, red color) patterning inside the PDMS chamber positioned on the device surface using (k) A_0_ mode (1.38 MHz, 0.3 W) and (l) S_0_ mode (4.86 MHz, 0.3 W). The scale bars in panels (e)–(h) are 500 μm, and the scale bars in panels (i)–(l) are 300 μm. The PDMS chamber is placed parallel to the IDT. The signs of minus and plus in the legend of the simulation represent the acoustic pressure direction, the termd “max” and “min” represent the maximum value of the acoustic pressure at the node and antinode.

We first investigated particle patterning within a droplet using the Lamb traveling wave. Previous study indicated that when the Lamb traveling wave was propagated into the droplet with a relatively large size (e.g., larger than 5 μL), a standing wave field was generated within the droplet due to the reflection at the droplet boundary.^63^ The standing wave field then produced a periodic acoustic pressure field within the droplet, thereby generating various particle patterns. Figures 4a–d show the simulated acoustic pressure field within the droplet positioned on the device surface using different orders of the Lamb traveling wave. Clearly, a checkerboard acoustic pressure field was formed under the actuation of different orders of Lamb wave. When the particle solution was placed on the device surface, under the actuation of an acoustic radiation force, the particles in the solution would accumulate at these pressure node areas, thereby forming the checkerboard patterns of particles (Movie S7), as shown in Figures 4e–h. As the acoustic wave mode was changed to a higher order or the resonant frequency of acoustic wave mode was increased, the size of the formed checkerboard patterns became much smaller, and the checkerboard patterns became irregular due to a weak acoustic field generated at higher-order modes. The results are consistent with the previous study.^63^Figures 4I and 4j show the simulated acoustic pressure fields inside the rectangular PDMS chamber placed on the device surface (in front of the IDT). When the Lamb traveling waves dissipated their energy into the PDMS chamber, the interference between the radiated traveling wave and the reflected acoustic wave would generate a standing wave field within the PDMS chamber,^24,33^ thereby forming linear pressure node patterns parallel to the PDMS channel walls as well as the IDT. When the particle solution was injected into the PDMS chamber, the particles would be accumulated on these pressure node lines and patterned into lines parallel to the IDTs (Movie S8), as shown in Figures 4k and 4l. As the frequency of the acoustic wave mode was increased, the distance between the adjacent lines was decreased. The simulation results show good agreement with the experiment ones.

**Figure 5:**
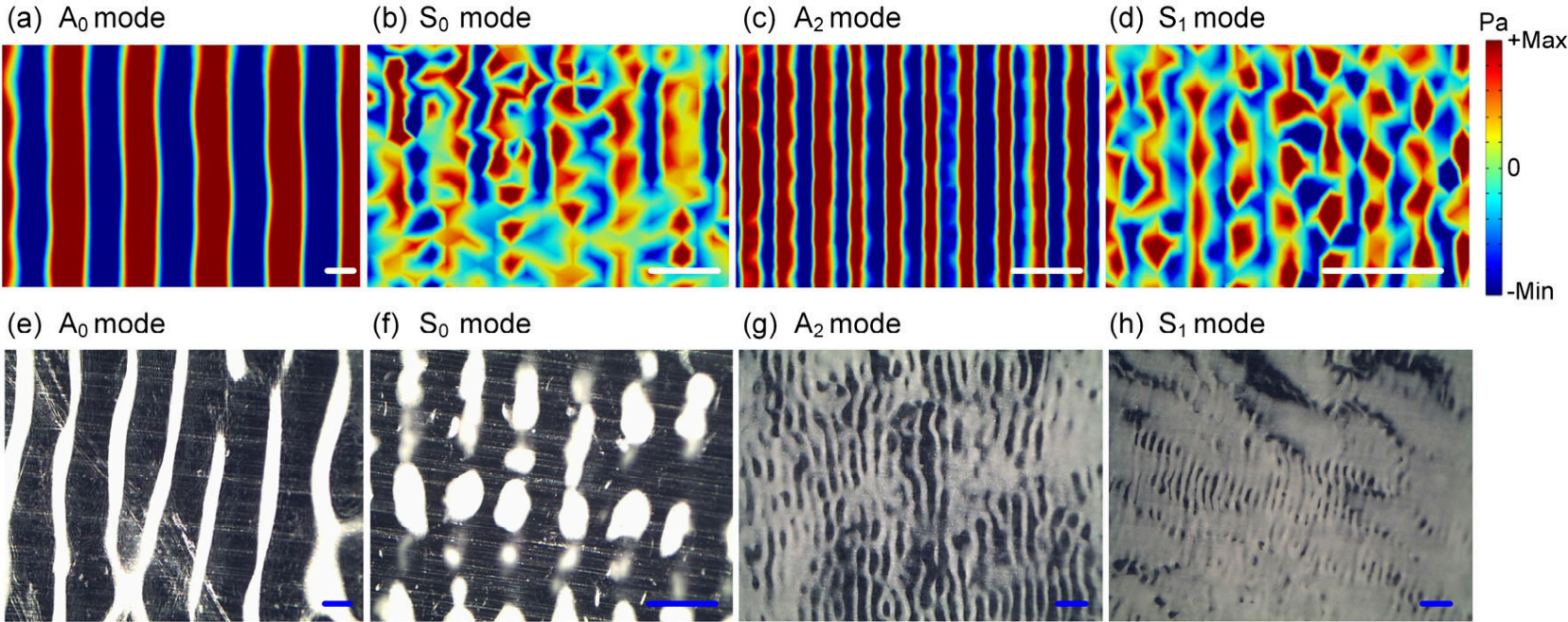
Simulation of acoustic pressure fields inside the PDMS chamber positioned at backside of Lamb wave device using different modes/orders of Lamb wave: (a) A_0_ mode (1.39 MHz), (b) S_0_ mode (4.81 MHz), (c) A_2_ mode (6.83 MHz), and (d) S_1_ mode (8.81 MHz). Silica particles (diameter of 5 μm, white color) patterning inside the PDMS chamber placed at the device backside using different modes/orders of Lamb traveling wave, (e) A_0_ mode (1.38 MHz, 0.3 W), (f) S_0_ mode (4.86 MHz, 0.5 W), (g) A_2_ mode (6.81 MHz, 0.5 W), and (h) S_1_ mode (8.84 MHz, 0.6 W). All scale bars are 200 μm. The PDMS chamber is positioned parallel to the IDT.

The Lamb wave is a plate wave, propagating through the whole substrate; thus, the backside of the Lamb wave device could also generate particle patterns. Here, we further investigated particle patterning behaviors within a PDMS chamber positioned at the backside of a Lamb wave device. Figures 5a–d show the simulated acoustic pressure fields inside the PDMS chamber at the device’s backside, which are similar to those observed on the top surface. Linear patterns of pressure nodes parallel to the PDMS channel walls and the IDT were clearly formed inside the PDMS chamber placed at the device backside, because the interference between the incident Lamb traveling wave and the reflected wave generated the standing wave. When the particle solutions were injected into the PDMS chamber, linear particle patterns parallel to the IDT were formed (Movie S9) at the pressure nodes, as illustrated in Figures 5e–h. The formed particle patterns at the backside of Lamb wave device are similar to those formed on the device’s top surface, whereas the particle patterning at the device backside needs a higher RF power due to a weaker acoustic field. However, we should address that acoustofluidic manipulation at the device’s backside reduces the potential contamination to the IDT electrodes and is convenient for cleaning. The distance between the adjacent lines is decreased with the increase of frequency of acoustic wave mode, as summarized in Figure S7, which is consistent with the previous study.^45^

**Figure 6:**
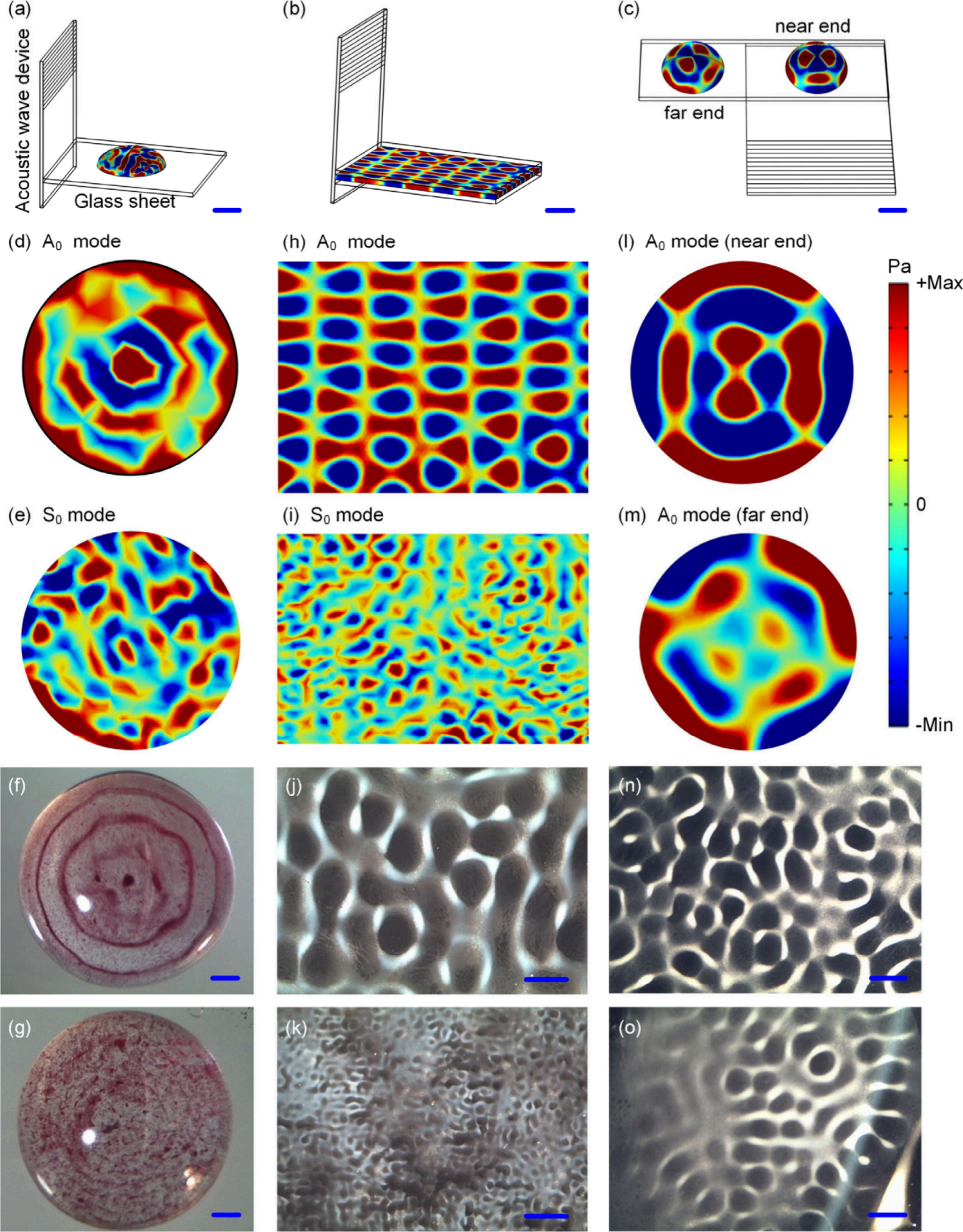
Schematic of experimental setup for generating particle patterns: (a) the droplet on top of the glass sheet (the glass sheet is positioned vertical to the acoustic wave device), (b) the liquid in the gap between two pieces of glass (two glass sheets are positioned vertical to the acoustic wave device), and (c) two droplets on the glass sheet (the glass sheet is placed parallel to the device surface). Simulation of acoustic pressure fields within the droplet positioned on top of the glass sheet was performed using (d) the A_0_ mode (1.39 MHz) and (e) the S_0_ mode (4.81 MHz). Polystyrene particle (diameter of 10 μm, red color) patterning within the droplet (50 μL) placed on top of the glass sheet was driven by (f) A_0_ mode (1.38 MHz, 1 W) and (g) S_0_ mode (4.86 MHz, 1 W). Simulation of acoustic pressure fields inside the liquid gap between two pieces of glass using the (h) A_0_ mode (1.39 MHz) and (i) S_0_ mode (4.81 MHz). Silica particle (diameter of 5 μm, white color) patterning inside the gap between two pieces of glass using (j) A_0_ mode (1.38 MHz, 1 W), (k) S_0_ mode (4.86 MHz, 1 W). Simulation of acoustic pressure fields within the droplet placed on the glass sheet (the glass sheet is placed parallel to the device surface) using A_0_ mode for (l) the sessile droplet near the acoustic wave device and (m) the sessile droplet far away from the acoustic wave device. Silica particle (diameter of 5 μm, white color) patterning within the sessile droplet placed on the glass sheet driven by the A_0_ mode (1.38 MHz, 1 W) for (n) the sessile droplet near the acoustic wave device and (o) the sessile droplet far away from the acoustic wave device. All scale bars are 500 μm.

We further studied particle patterning formed by putting a particle solution on the top of the glass or injecting into the gap between two pieces of glass, as illustrated in Figures 6a–c. Here, the acoustic wave was coupled into the glass using the ultrasonic gel. Figure S8 shows that when the acoustic wave is propagated into the glass (positioned vertical to the acoustic wave device), the generated acoustic wave in the glass is also the Lamb wave. However, the acoustic wave vibration in the glass is influenced by the loaded droplet; for example, a local circular vibration region on the glass is observed at the placed position of the droplet. Figures 6d and 6e show simulated acoustic pressure fields within the droplet positioned on the glass sheet under the actuation of the A_0_ mode and S_0_ mode of the Lamb wave, respectively. Here, the glass sheet was positioned vertically to the acoustic wave device, as illustrated in Figure 6a. It has been shown that when the Lamb wave propagating on the glass was coupled into the droplet, a standing wave field was formed within the droplet, thereby generating annular pressure node patterns. Under the actuation of acoustic radiation force, the particles in solution were accumulated on these pressure node rings, thereby forming the annular patterns of particles (Movie S10), as shown in Figures 6f and 6g. Changing the acoustic wave modes or frequencies caused changes in sizes or intervals between adjacent annular patterns.

When the particle solution was injected into the gap between two pieces of glass, as shown in Figure 6b, the faveolate particle patterns were formed (Movie S11), as shown in Figures 6j and 6k. Here, the white regions represent the aggregated particles, while the dark regions represent the surface of glass sheet. These have also been verified through FEA simulation of acoustic pressure fields, as shown in Figures 6h and 6i. The faveolate particle patterns could also be formed by putting a drop of particle solution on the glass sheet that was placed parallel to the device surface, as shown in Figures 6l–o. Therefore, for particle patterning functions induced by multimode acoustic waves, we can adjust the particle pattern sizes or shapes by either varying the frequency of acoustic wave mode or changing the experimental configurations. In addition, for particle patterning on the glass, this superstrate type of design avoids the contamination of the acoustic wave device by liquid samples, thereby enabling the reuse of acoustic wave devices. All of these results show that multimode acoustofluidic technologies have great potential in biomedical applications such as diversified particles/cell patterning, focusing, trapping, and solidified particle–hydrogel patterns.

## 4 Conclusions

In summary, we have achieved various acoustic wave mode excitations on piezoelectric ZnO-thin-film-coated aluminum sheets by varying the ratios between IDT pitch/wavelength and substrate thickness. Based on these acoustic wave modes, we systematically investigated fluidic actuation and particle patterning behaviors, demonstrating acoustofluidic diversity using multiple modes of acoustic waves. We provided a guidance for high-performance acoustofluidic actuation platform design by demonstrating that the Rayleigh mode, hybrid mode (e.g., pseudo-Rayleigh mode and A_0_ mode) generally have better fluidic actuation performance than those of the Sezawa mode, symmetric mode (e.g., S_0_ and S_1_) and higher-order modes of the Lamb wave. Also, we demonstrated diversified particle patterning generation either on both sides of the Lamb wave device or on the glass sheet (by coupling of acoustic wave into the glass using the gel) by varying the frequency of the acoustic wave mode and experimental configurations. The formation mechanisms of particle patterning were analyzed through the FEA simulation of acoustic pressure fields.

## Supporting Information Available

Droplet (1 μL) pumping using ZnO thin film acoustic wave device fabricated on the 600-μm-thick Al sheet (wavelength of 200 μm) substrate with the Rayleigh wave mode and an input RF power of 1.5 W (Movie S1) (AVI)
Droplet (1 μL) pumping using ZnO thin film acoustic wave device fabricated on a 600-μm-thick Al sheet substrate (wavelength of 200 μm) with the Sezawa mode and an input RF power of 21 W (Movie S2) (AVI)
Droplet (1 μL) pumping using ZnO thin film acoustic wave device fabricated on a 200-μm-thick Al sheet substrate (wavelength of 400 μm) with the A_0_ mode and an input RF power of 0.8 W (Movie S3) (AVI)
Droplet (1 μL) pumping using ZnO thin film acoustic wave device fabricated on a 200-μm-thick Al sheet substrate (wavelength of 400 μm) with the S_1_ mode and an input RF power of 3 W (Movie S4) (AVI)
Droplet (8 μL) pumping using ZnO thin film acoustic wave device fabricated on a 200-μm-thick Al sheet substrate (wavelength of 1100 μm) with the A_0_ mode and an input RF power of 10 W (Movie S5) (AVI)
Droplet (5 μL) pumping using ZnO thin film acoustic wave device fabricated on a 200-μm-thick Al sheet substrate (wavelength of 1100 μm) with the A_0_ mode and an input RF power of 10 W (Movie S6) (AVI)
Polystyrene particle (diameter of 10 μm) patterning within the droplet (50 μL) positioned on the device surface using the A_0_ mode (wavelength of 1100 μm) and an input RF power of 0.3 W (Movie S7) (AVI)
Polystyrene particle (diameter of 10 μm) patterning within the PDMS chamber positioned on the device surface using the S_0_ mode (wavelength of 1100 μm) and an input RF power of 0.3 W (Movie S8) (AVI)
Silica particle (diameter of 5 μm) patterning within the PDMS chamber positioned on the device backside using the A_0_ mode (wavelength of 1100 μm) and an input RF power of 0.5 W (Movie S9) (AVI)
Polystyrene particle (diameter of 10 μm) patterning within the droplet (50 μL) positioned on the top of glass sheet using the A_0_ mode (wavelength of 1100 μm) and an input RF power of 1 W (Movie S10) (AVI)
Silica particle (diameter of 5 μm) patterning inside the gap between two pieces of glass using the A_0_ mode (wavelength of 1100 μm) and an input RF power of 1 W (Movie S11) (AVI)
XRD pattern and cross-sectional SEM image of ZnO thin film on a 200-μm-thick Al sheet; optical images of the fabricated acoustic wave devices; schematic of the model and meshing of the model for simulation of acoustic wave mode and acoustic pressure field; schematic of experimental setup for fluid actuation; FEA simulation of acoustic wave mode and experimental measured reflection spectra for ZnO thin film acoustic wave device with wavelengths varied from 100 μm to 400 μm fabricated on 600-μm-thick Al sheets; FEA simulation of acoustic wave modes and experimental measured reflection spectra for ZnO thin film acoustic wave device with wavelengths varied from 100 μm to 1100 μm fabricated on 200-μm-thick Al sheets; distance (μm) between the adjacent lines as a function of acoustic wave mode frequency; FEA simulation of acoustic vibration on the acoustic wave device and on the glass sheet (PDF)

## Supplementary Material

am4c06480_si_001.avi

am4c06480_si_002.avi

am4c06480_si_003.avi

am4c06480_si_004.avi

am4c06480_si_005.avi

am4c06480_si_006.avi

am4c06480_si_007.avi

am4c06480_si_008.avi

am4c06480_si_009.avi

am4c06480_si_010.avi

am4c06480_si_011.avi
