# Supplementary material for: Acoustofluidic Diversity Achieved by Multiple Modes of Acoustic Waves Generated on Piezoelectric-Film-Coated Aluminum Sheets
Source: ACS Appl Mater Interfaces. 2024 Aug 15;16(34):45119–30. doi: 10.1021/acsami.4c06480 (PMC11367575; doi:10.1021/acsami.4c06480)
Supplement: Supplementary file 12 — am4c06480_si_012.pdf [file am4c06480_si_012.pdf]

Supporting Information

Acoustofluidic diversity achieved by multiple modes of acoustic waves generated on piezoelectric film coated aluminum sheets

Yong Wang[a,b,c,†], Xianbin Li[d,†], Hui Meng[a], Ran Tao[e,c], Jingui Qian[d], Chen Fu[e], Jingting Luo[e], Jin Xie[b,*], and Yongqing Fu[c,*]

[a] Department of Mechanical Engineering, Hangzhou City University, Hangzhou 310015, China

[b] The State Key Laboratory of Fluid Power and Mechatronic Systems, Zhejiang University, Hangzhou 310027, China

[c] Faculty of Engineering and Environment, University of Northumbria, Newcastle upon Tyne NE1 8ST, UK

[d] Anhui Province Key Laboratory of Measuring Theory and Precision Instrument, School of Instrument Science and Opto-Electronics Engineering, Hefei University of Technology, Hefei 230009, China

[e] Shenzhen Key Laboratory of Advanced Thin Films and Applications, College of Physics and Optoelectronic Engineering, Shenzhen University 518060, China

[†] These authors contributed equally to this paper.

* Corresponding authors: xiejin@zju.edu.cn; richard.fu@northumbria.ac.uk

Figure

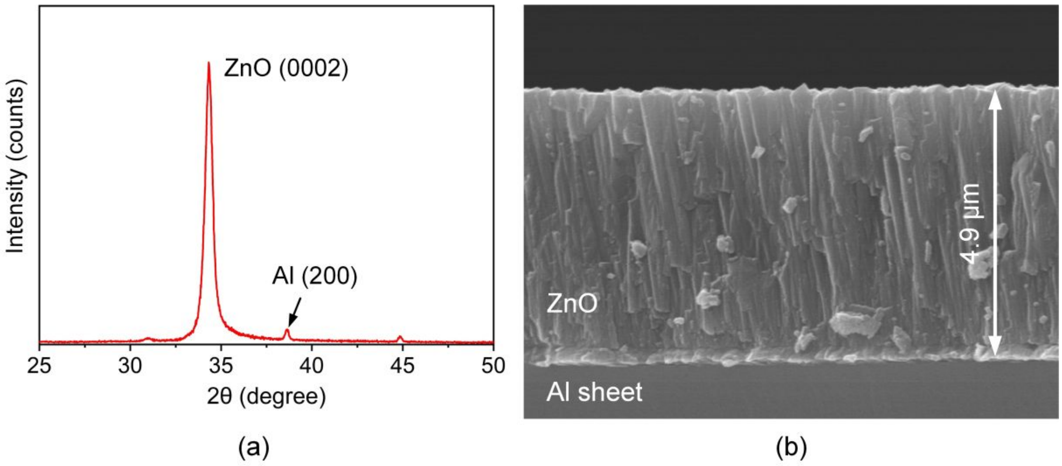

Figure S1. (a) XRD pattern of ZnO thin film on 200 μm thick Al sheet substrate. (b) Cross-section SEM image of ZnO thin film on 200 μm thick Al sheet substrate, indicating a columnar morphology of ZnO microstructure.

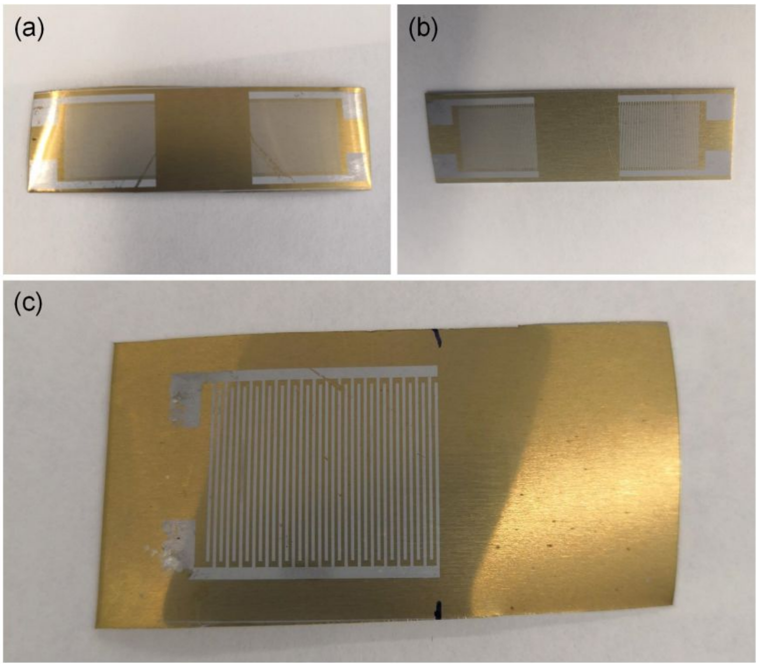

Figure S2. Optical images of the fabricated ZnO thin film acoustic wave devices on (a) 200 μm thick Al sheet substrate with wavelength of 200 μm, (b) 600 μm thick Al sheet substrate with wavelength of 200 μm and (c) 200 μm thick Al sheet substrate with wavelength of 1100 μm.

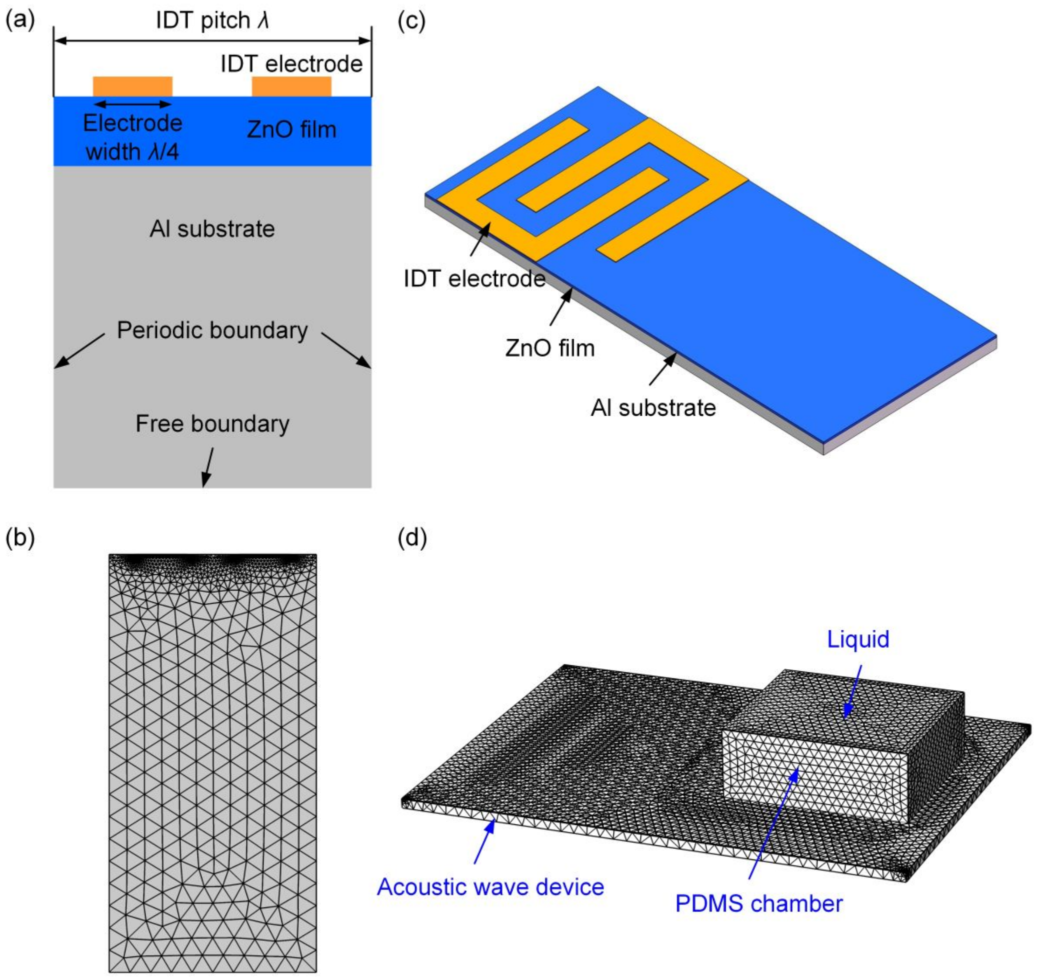

Figure S3. (a) Schematic of 2D model used in the simulation. (b) Meshing of 2D model for simulation of acoustic wave mode. (c) Schematic of 3D model of Lamb wave device. (d) Meshing of 3D model for simulation of acoustic pressure field within the PDMS chamber.

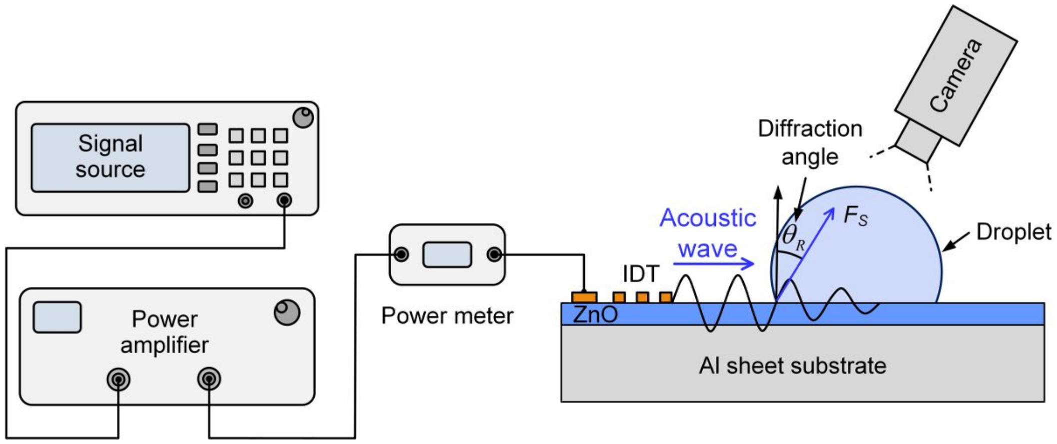

Figure S4. Schematic of experimental setup for fluid actuation test. At a RF power of a few watts, the droplet can be actuated forward by the acoustic wave.

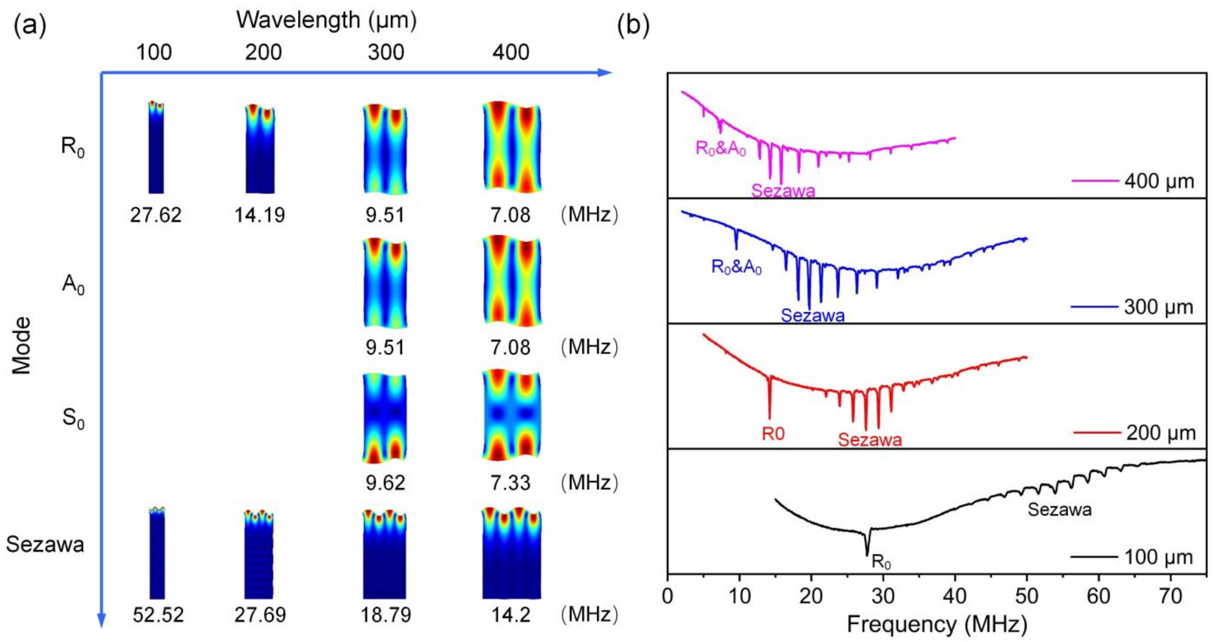

Figure S5. (a) FEA simulation results of acoustic wave vibration modes and (b) the measured reflection spectra for ZnO thin film acoustic wave devices fabricated on 600 μm-thick Al sheet substrate with wavelengths varied from 100 to 400 μm.

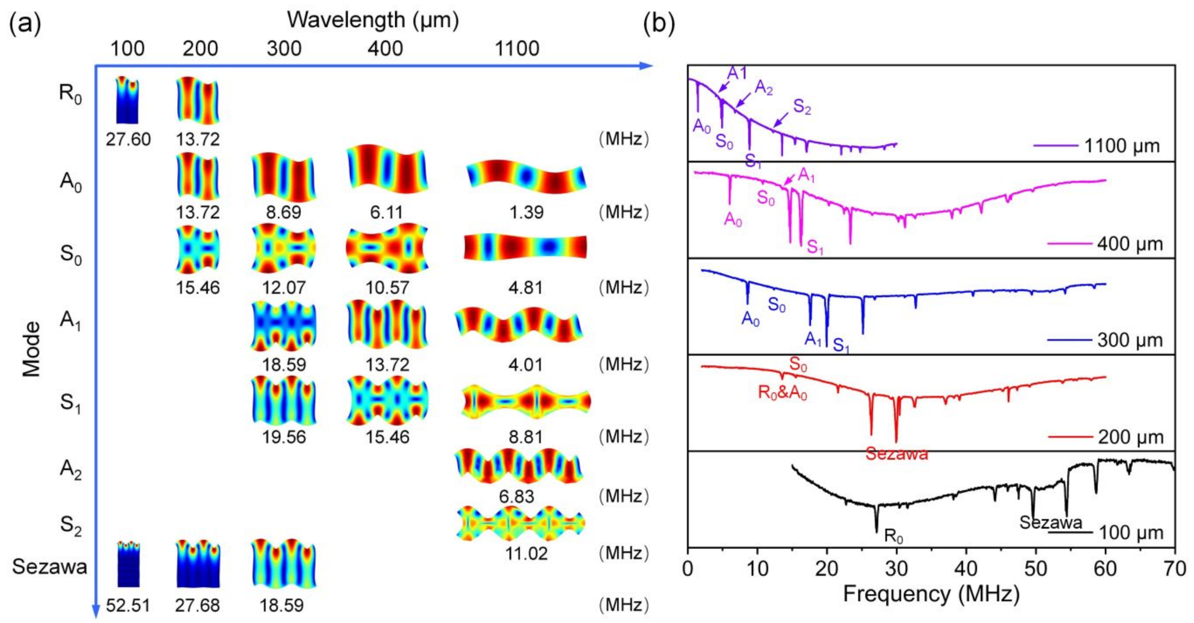

Figure S6. (a) FEA simulation results of acoustic wave vibration modes and (b) the measured reflection spectra for ZnO thin film acoustic wave devices fabricated on 200 μm-thick Al sheet substrate with wavelengths varied from 100 to 1100 μm.

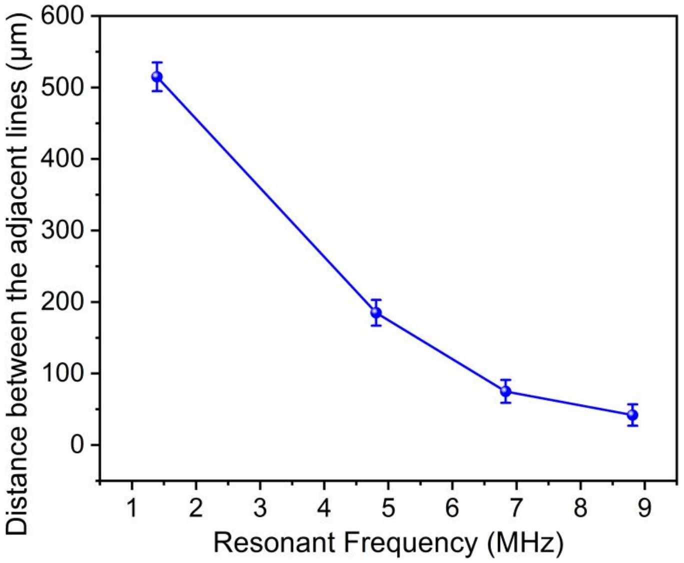

Figure S7. Distance (μm) between the adjacent lines as a function of acoustic wave mode frequency.

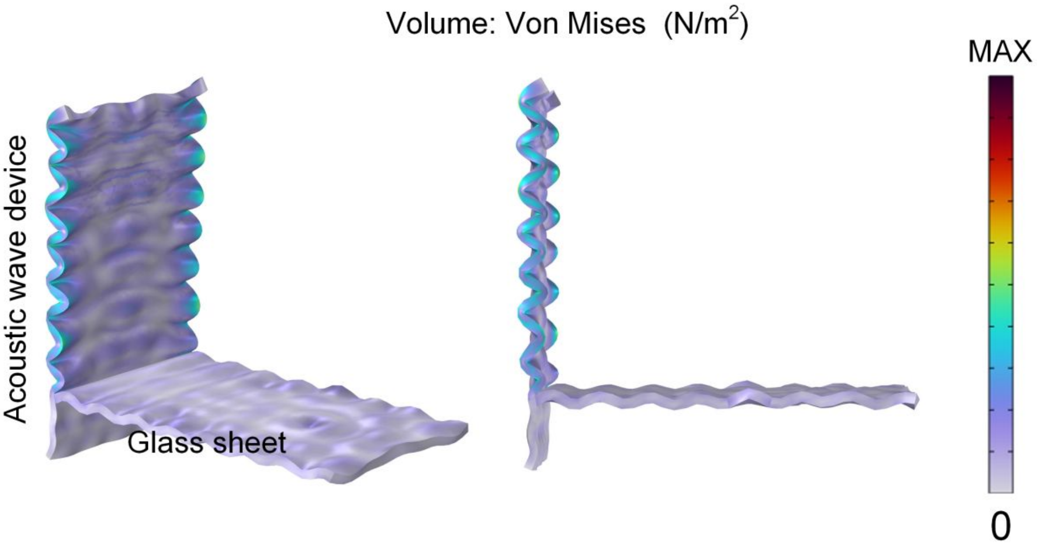

Figure S8. FEA simulation of acoustic wave vibration on the acoustic wave device and on the glass sheet using the A_0 mode. The acoustic wave is coupled into the glass from the acoustic wave device using the ultrasonic gel.

Movie

Movie S1. Droplet (1 μL) pumping using ZnO thin film acoustic wave device fabricated on 600 μm thick Al sheet (wavelength of 200 μm) substrate with the Rayleigh wave mode and an input RF power of 1.5 W.

Movie S2. Droplet (1 μL) pumping using ZnO thin film acoustic wave device fabricated on 600 μm thick Al sheet substrate (wavelength of 200 μm) with the Sezawa mode and an input RF power of 21 W.

Movie S3. Droplet (1 μL) pumping using ZnO thin film acoustic wave device fabricated on 200 μm thick Al sheet substrate (wavelength of 400 μm) with the A_0 mode and an input RF power of 0.8 W.

Movie S4. Droplet (1 μL) pumping using ZnO thin film acoustic wave device fabricated on 200 μm thick Al sheet substrate (wavelength of 400 μm) with the S_1 mode and an input RF power of 3 W.

Movie S5. Droplet (8 μL) pumping using ZnO thin film acoustic wave device fabricated on 200 μm thick Al sheet substrate (wavelength of 1100 μm) with the A_0 mode and an input RF power of 10 W.

Movie S6. Droplet (5 μL) pumping using ZnO thin film acoustic wave device fabricated on 200 μm thick Al sheet substrate (wavelength of 1100 μm) with the A_0 mode and an input RF power of 10 W.

Movie S7. Polystyrene particle (diameter of 10 μm) patterning within the droplet (50 μL) positioned on the device surface using the A_0 mode (wavelength of 1100 μm) and an input RF power of 0.3 W.

Movie S8. Polystyrene particle (diameter of 10 μm) patterning within the PDMS chamber positioned on the device surface using the S_0 mode (wavelength of 1100 μm) and an input RF power of 0.3 W.

Movie S9. Silica particle (diameter of 5 μm) patterning within the PDMS chamber positioned on the device backside using the A_0 mode (wavelength of 1100 μm) and an input RF power of 0.5 W.

Movie S10. Polystyrene particle (diameter of 10 μm) patterning within the droplet (50 μL) positioned on the top of glass sheet using the A_0 mode (wavelength of 1100 μm). The glass sheet is positioned vertical to the acoustic wave device. The acoustic wave is coupled into the glass using the ultrasonic gel with an input power of 1 W. The rotation of circular particle patterns or droplet shake is caused by the acoustic streaming effect at a high RF power.

Movie S11. Silica particle (diameter of 5 μm) patterning inside the gap between two pieces of glass using the A_0 mode (wavelength of 1100 μm). Two glass sheets are positioned vertical to the acoustic wave device. The acoustic wave is coupled into the glass using the ultrasonic gel with an input power of 1 W.
